# Asymmetric Dimethylarginine Blocks Nitric Oxide-Mediated Alcohol-Stimulated Cilia Beating

**DOI:** 10.1155/2013/592892

**Published:** 2013-11-06

**Authors:** T. A. Wyatt, S. M. Wells, Z. A. Alsaidi, J. M. DeVasure, E. B. Klein, K. L. Bailey, J. H. Sisson

**Affiliations:** ^1^VA Nebraska-Western Iowa Health Care System Research Service, Department of Veterans Affairs Medical Center, 4101 Woolworth Avenue, Omaha, NE 68105, USA; ^2^Department of Environmental, Agricultural, and Occupational Health, College of Public Health, University of Nebraska Medical Center, Omaha, NE 68198, USA; ^3^Pulmonary, Critical Care, Sleep & Allergy Division, Department of Internal Medicine, 985300 Nebraska Medical Center, Omaha, NE 68198-5300, USA

## Abstract

The airway epithelium is exposed to alcohol during drinking through direct exhalation of volatized ethanol from the bronchial circulation. Alcohol exposure leads to a rapid increase in the cilia beat frequency (CBF) of bronchial epithelial cells followed by a chronic desensitization of cilia stimulatory responses. This effect is governed in part by the nitric oxide regulation of cyclic guanosine and adenosine monophosphate-dependent protein kinases (PKG and PKA) and is not fully understood. Asymmetric dimethylarginine (ADMA), an endogenous inhibitor of nitric oxide synthase, is implicated in the pathogenesis of several pulmonary disorders. We hypothesized that the inhibition of nitric oxide synthase by ADMA blocks alcohol-stimulated increases in CBF. To test this hypothesis, ciliated primary bovine bronchial epithelial cells (BBEC) were preincubated with ADMA (100 **µ**M) and stimulated with 100 mM ethanol. CBF was measured and PKA assayed. By 1 hr, ethanol activated PKA, resulting in elevated CBF. Both alcohol-induced PKA activation and CBF were inhibited in the presence of ADMA. ADMA alone had no effect on PKA activity or CBF. Using a mouse model overexpressing the ADMA-degrading enzyme, dimethylarginine dimethylaminohydrolase (DDAH), we examined PKA and CBF in precision-cut mouse lung slices. Alcohol-stimulated increases in lung slice PKA and CBF were temporally enhanced in the DDAH mice versus control mice.

## 1. Introduction

Alcohol use disorders are associated with increased lung infections including pneumonia and bronchitis [1]. While alcohol is an established risk factor for acute lung injury leading to sepsis [2], lung immunosuppression is also a common response to heavy and sustained alcohol use [3]. Chronic alcohol exposure also impairs lung innate host defense by desensitizing effective mucociliary clearance [1], rendering cilia unresponsive to stress. Collectively, this multifaceted impact of alcohol on the pathophysiology of lung function has been termed “alcoholic lung disease” [4].

Alcohol is a small, uncharged molecule that readily crosses the lipid bilayer of all cell membranes and easily enters the lungs, where as much as 15% of ingested alcohol can be excreted by the exhalation of volatilized alcohol through the airways. In contrast to dissolved oxygen and carbon dioxide, which traffic through the pulmonary circulation to and from the alveolar spaces, systemic arterial blood alcohol directly off-gases into the conducting airways via diffusion from the bronchial circulation [5]. Furthermore, higher localized concentrations of ethanol can be found in the airways of the lungs due to vapor condensation or “rain” effect of exhaled alcohol [1]. For these reasons, the ciliated airway epithelial cells lining the lungs are directly exposed to significant amounts of alcohol in persons who drink. 

When ciliated airway epithelial cells are exposed to pathophysiologic concentrations of ethanol for extended periods of time, a change occurs in the cells' ability to respond to external stimuli. Specifically, the normative cilia stimulatory response to inhaled particles of increased beat frequency becomes desensitized with sustained alcohol exposure [1]. This desensitization involves the uncoupling of downstream cyclic nucleotide-dependent protein kinase action on ciliary axonemal targets [6]. In contrast, brief alcohol exposure rapidly and transiently increases cilia beat frequency (CBF) of bronchial epithelial cells [7]. This alcohol effect on cilia is mediated by the nitric oxide (NO)-mediated regulation of cyclic adenosine monophosphate-dependent protein kinase (PKA) [1]. However, the exact mechanism that transitions the initial alcohol-induced cilia stimulatory response toward eventual cilia desensitization is not fully understood.

First demonstrated over 20 years ago [8], significant levels of ADMA are found in the lung [9] and specifically the bronchial epithelium [10]. Dysregulation of ADMA has been implicated in the pathogenesis of several pulmonary disorders such as asthma, fibrosis, pulmonary hypertension, and sepsis [11]. Previously, we have demonstrated that exogenously applied synthetic inhibitors of NOS are capable of blocking alcohol-stimulated PKA activation, and thereby preventing the stimulated CBF response [1, 12]. Therefore, we hypothesized that the action of an endogenously produced nitric oxide synthase inhibitor, ADMA, blocks alcohol-stimulated increases in CBF. The action of ADMA on NOS could therefore represent the mechanistic pathway in the alcohol-mediated transition of cilia from stimulatory to desensitized phenotype.

## 2. Materials and Methods

### 2.1. Mice

Wild type (WT) C57BL/6 and dimethylarginine dimethylaminohydrolase transgenic (DDAH-I) mice on the C57BL/6 strain that overexpress DDAH were originally obtained from The Jackson Laboratory (Bar Harbor, ME) and subsequently bred and maintained in microisolator units in the UNMC specific pathogen-free animal facility. The transgene expression of hDDAH is observed in aorta, heart, and brain. Mice were allowed food and water *ad libitum *and were used experimentally at 6–12 weeks of age. All animals were used in accordance with National Institutes of Health guidelines and the University of Nebraska Medical Center Institutional Animal Care, and Use Committee approved the study. 

### 2.2. *Ex Vivo* Mouse Tracheal Ring Model

Mice were sacrificed, and tracheae and lungs were removed. Each trachea was removed and maintained in sterile serum-free M199 containing penicillin/streptomycin (100 units/100 mg per mL) (Gibco) and fungizone (2 *μ*g/mL) (Gibco) at room temperature. Tracheal rings were cut (width *≈* 0.5–1 mm) from the distal end of the trachea just proximal to the first bifurcation of the trachea into right and left mainstream bronchi. The rings were incubated in serum-free M199 (Gibco) for 30 min prior to measuring CBF determinations. After measuring baseline CBF, tracheal rings were incubated with experimental treatments for up to 6 hr at 37°C and 5% CO_2_ then were allowed to equilibrate at 25°C for 10 min before CBF readings were recorded. In addition, the posttreatment epithelial cells from the remaining trachea were extracted with a sterile cell lifter (Fisher, Springfield, NJ) into cell lysis buffer as previously described [13]. The epithelial lysate was then immediately flash-frozen in liquid nitrogen for PKA activity assay.

### 2.3. *Ex Vivo* Mouse Lung Slice Model

Precision-cut lung (PCL) slices were made as previously described [14]. Briefly, C57BL/6 or DDAH-I mice were sacrificed and the lungs were inflated with low melting point agarose (Invitrogen, Carlsbad, CA). Chilled lungs were then sliced into 150 *μ*m precision-cut slices (OTS 4500 Tissue slicer, Electron Microscopy Sciences, Hatfield, PA) followed by incubation at 37°C to remove the agarose. Slices (3–5 per well) were placed in 12-well tissue culture plates. After 5 days of incubation with daily change of media, slices were exposed for up to 1 hr with 100 mM ethanol, and CBF was measured. Lung slices were snap-frozen in liquid nitrogen for PKA activity assay. Data were normalized to the total amount of PCL protein contained in each well. 

### 2.4. Ciliated Cell Culture

Ciliated mouse tracheal epithelial cells (MTEC) were cultured using an air-liquid interface system as previously described [15]. Primary bovine bronchial epithelial cells (BBEC) were obtained fresh from cow lungs (ConAgra Inc., Omaha, NE) and grown to confluent monolayers in culture as previously described [16]. 

### 2.5. Cell Viability Assay

An aliquot (50 *μ*L) of supernatant media from BBEC monolayers, tracheal rings, MTEC, or PCL slices treated with ADMA, ethanol, or media alone was assayed for cell viability using a TOX-7 kit (Sigma) to measure lactate dehydrogenase (LDH) release, according to the manufacturer's instructions. As a positive control, confluent 60 mm dishes of BBEC cells were lysed and LDH was measured.

### 2.6. Immunohistochemistry for ADMA and DDAH

Mouse lungs were inflation-fixed through the trachea with 10% formaldehyde-PBS, processed, paraffin-embedded, and sectioned (5 *μ*m). Mounted slides were either stained for ADMA or DDAH expression. Immunohistochemical detection of free and protein-bound ADMA was performed using a rabbit anti-ADMA antibody (1 : 1000; EMD Millipore, Billerica, MA) or rabbit anti-DDAH-I and DDAH-II antibodies (1 : 400; Santa Cruz Biotechnology, Dallas, TX). Sections were deparaffinized, hydrated, and washed with PBS. Before staining, endogenous peroxidase activity was inhibited using Peroxo-Block (Zymed Laboratories, South San Francisco, CA). Slides were incubated for 30 min with primary antibody and developed using an immunoperoxidase kit (Vector Laboratories, Burlingame, CA). Control sections were incubated with a nonspecific normal rabbit IgG (Sigma) followed by secondary antibody. All sections were counterstained with hematoxylin (Fisher Scientific). All slides were scanned by the Tissue Science Facility at the University of Nebraska Medical Center on a Ventana Coreo at 40x with a resolution of 0.2325 microns per pixel (Ventana, Tucson, Arizona).

### 2.7. Cilia Beat Frequency Assay

CBF was recorded from adhered cells, tracheal rings, and precision-cut lung slices in media-submerged cultures. The frequency of the beating cilia and the total number of motile points within a given field of view were determined using the Sisson-Ammons Video Analysis (SAVA) system [17].

### 2.8. PKA Activity Assay

Primary MTEC isolated from trachea directly or cultured on air-liquid interface as well as PCL slices was assayed for PKA activity. After experimental treatment conditions, culture supernatants were removed, 250 *μ*L of cell lysis buffer was added, and cells or tissue was flash-frozen. Dishes were thawed and scraped into centrifuge tubes and kept on ice. The supernatant containing the cells was sonicated, and cells were centrifuged at 10,000 ×g at 4°C for 30 min. PKA activity was measured in the soluble fraction from the extracted cell or tissue sample as previously described [13]. Data was standardized to the total amount of cell protein assayed and expressed as pmol radiolabeled phosphate transferred onto a standard amount of heptapeptide substrate (Leu-Arg-Arg-Ala-Ser-Leu-Gly; Sigma) per minute of reaction time. Each unique condition was measured a minimum of 3 separate experiments. 

### 2.9. Statistical Analysis

Replicate data from at least 3 separate experiments are presented as the mean ± standard error of mean (SEM). One-way analysis of variance (ANOVA) with Tukey multicomparison posttest was employed to compare responses between 3 or more groups. Differences between groups were accepted as significant using a 95% confidence interval (*P* < 0.05). In all analyses, GraphPad Prism (San Diego, CA; version 5.01) software was utilized to determine statistical significance.

## 3. Results

### 3.1. ADMA and DDAH Are Located in the Ciliated Cells of the Airway Epithelium

**Figure 1:**
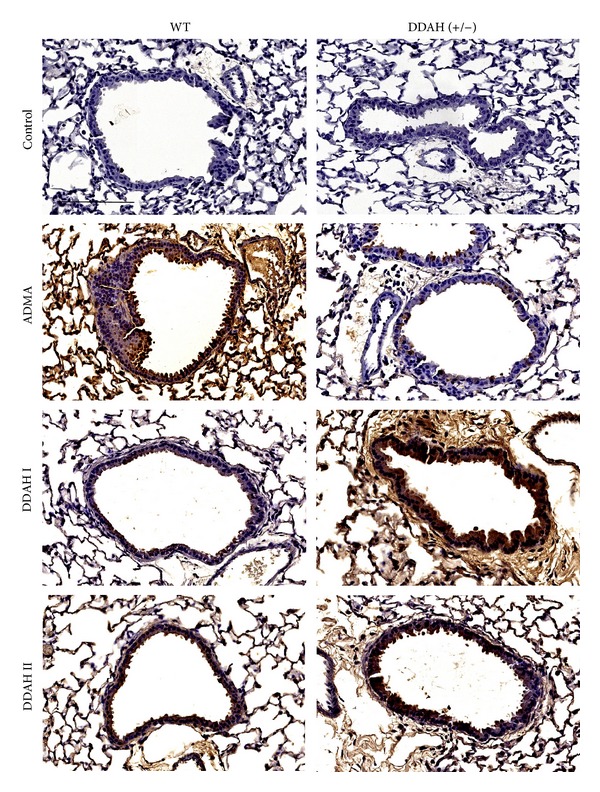
ADMA and DDAH are located in the ciliated cells of the airway epithelium. Free and bound ADMA, DDAH-I, and DDAH-II were immunolocalized in wild type (WT) and DDAH-I overexpressing [DDAH (+/−)] mouse lung tissue sections and visualized by immunoperoxidase stain. Controls consist of non-specific IgG primary antibody. Original magnification is 40x. Bar represents 100 *μ*m.

While the lung has been shown to have a high expression of DDAH-I [18] and DDAH-II [10], specific airway epithelial cell expression of ADMA has not been demonstrated. We hypothesized that ADMA and DDAH are preferentially expressed in ciliated airway cells. To test this hypothesis, tissue immunohistochemistry was performed on lung sections from mice to determine the presence and cellular location of both ADMA and DDAH. Sections were stained with primary antibodies to ADMA, DDAH-I, or DDAH-II and detected using a secondary immunoperoxidase-conjugated antibody detection reaction. Readily visible brown staining was observed in all lung sections corresponding to ADMA, DDAH-I, and DDAH-II in wild type mice (Figure 1). Staining of all 3 proteins was evident throughout the alveolar parenchyma but was particularly prominent in the epithelial cells lining the airways both in proximal and distal regions of the lung. Similarly, ADMA, DDAH-I, and DDAH-II were each detected in the lungs of transgenic DDAH-I expressing mice although the levels of ADMA appeared to be somewhat decreased, yet still present. All lung sections were counterstained with hematoxylin stain revealing visible purple stain in the absence of any positive brown staining due to primary antibody localization. As a control, lung tissue slices were incubated with a non-specific IgG followed by the same secondary antibody used above. These results demonstrate that the ciliated airway epithelium contains significant amounts of ADMA, DDAH-I, and DDAH-II.

### 3.2. Increasing ADMA Levels Alone Has No Baseline Effect on Cilia Beat

**Figure 2:**
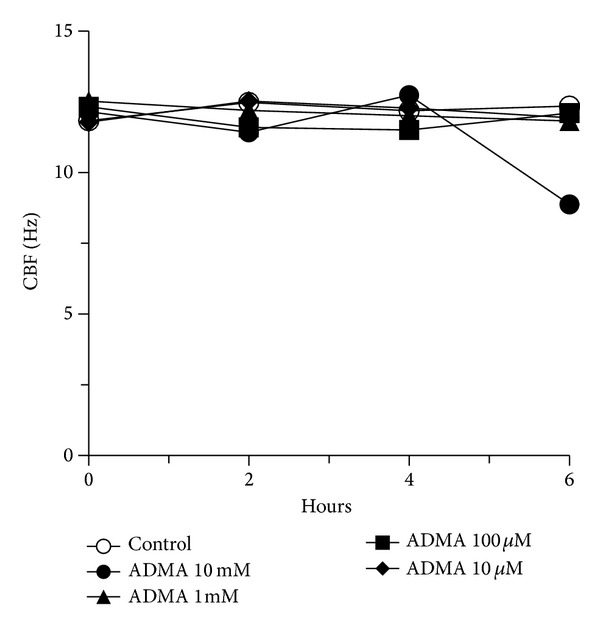
ADMA blocks alcohol-stimulated increases in both CBF and PKA activity in bovine bronchial epithelial cells. Cilia beat frequency (CBF) in ciliated mouse tracheal rings treated with 10 *μ*M–10 mM asymmetric dimethylarginine (ADMA) for up to 6 hr. ADMA had no effect on baseline CBF versus control media at any time point. Bars represent SEM of triplicate independent experiments (*n* = 3 mice) measuring at least 10 separate fields per experiment.

Because ADMA and DDAH were distinctly localized to the ciliated airway epithelium in the unstimulated state, we hypothesized that baseline unstimulated CBF is not NO-dependent so it would not be affected by changing ADMA levels. To test this hypothesis, we measured unstimulated CBF following exposure to supplemental ADMA. Mouse tracheal ring cilia beating was unaffected by exogenous treatment with 10 *μ*M–10 mM ADMA tested over a 6 hr period (Figure 2). A small, but insignificant, decrease in CBF at 6 hr treatment was observed under conditions of nonphysiologic concentrations (10 mM) of ADMA. Similarly, baseline unstimulated CBF in tracheal rings from DDAH transgenic mice does not differ from that of wild type mice while beta agonist (procaterol; 10 nM) significantly stimulated CBF in both wild type and DDAH mice (data not shown). These data demonstrate that the increases in ADMA levels that are physiologically relevant do not alter cilia beating in the absence of any other cilia modulator.

### 3.3. ADMA Blocks Alcohol-Stimulated Increases in Both CBF and PKA Activity in Bovine Bronchial Epithelial Cells

**Figure 3:**
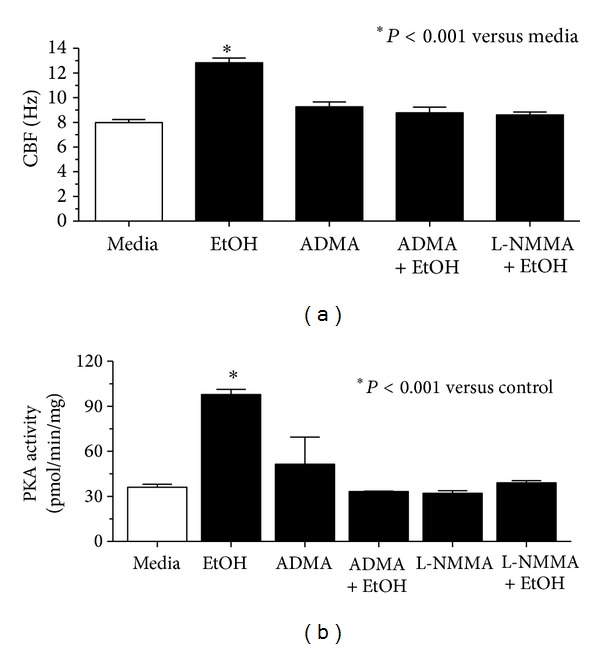
ADMA blocks alcohol-stimulated increases in both CBF and PKA activity in bovine bronchial epithelial cells. (a) Cilia beat frequency (CBF) in primary bovine bronchial epithelium treated with 100 mM alcohol (EtOH). Pretreatment (30 min) with ADMA (100 *μ*M) blocked 1 hr alcohol stimulation of CBF (*P* < 0.001) versus control media. This inhibition was comparable to that observed with the NOS blocker (L-NMMA; 10 *μ*M). (b) ADMA pretreatment blocks alcohol-stimulated increases in PKA activity in ciliated bovine epithelium (*P < 0.001). Bars represent SEM of triplicate separate experiments (*n* = 3).

Previously, we have shown that short-term alcohol treatment of ciliated bronchial epithelial cells rapidly and transiently stimulates increased cilia beating in a nitric oxide-dependent manner [1]. Although increasing ADMA by itself has no effect on CBF (as shown in Figure 2), we hypothesized that elevated ADMA would block the alcohol/NO-induced stimulation of CBF. Ciliary beat in primary cultures of ciliated bovine bronchial epithelial cells (BBEC) was significantly stimulated by 1 hr alcohol (100 mM; Figure 3(a)). Pretreatment of BBEC with 100 *μ*M ADMA for 30 min prior to alcohol stimulation completely blocked alcohol-induced increases in CBF. This inhibition of alcohol-stimulated CBF was comparable to that of the NOS inhibitor, NG-Monomethyl-L-arginine (L-NMMA). Because alcohol-stimulated NO production is required for alcohol-stimulated PKA activation and subsequent increases in CBF [1], we hypothesized that ADMA blocks PKA activation by alcohol. To test this hypothesis, we measured PKA activity in alcohol-exposed BBEC in the presence or absence of ADMA. While 1 hr alcohol (100 mM) treatment significantly elevated BBEC PKA, pretreatment with 100 *μ*M ADMA or 10 *μ*M L-NMMA for 30 min prior to alcohol stimulation blocked ethanol-stimulated increases in PKA (Figure 3(b)). These data show that ADMA prevents alcohol-stimulated and NO-mediated increases in PKA-stimulated CBF.

### 3.4. Alcohol Stimulation of Tracheal Epithelial CBF Is Enhanced in Mice Expressing an ADMA-Degrading Enzyme

**Figure 4:**
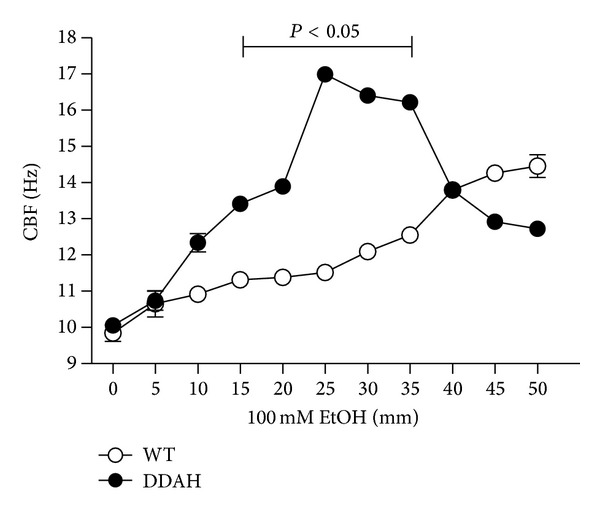
Alcohol stimulation of tracheal epithelial CBF is enhanced in mice expressing an ADMA-degrading enzyme. Tracheal rings from mice overexpressing the ADMA-degrading enzyme dimethylaminohydrolase (DDAH) were treated with 100 mM alcohol (EtOH) and their CBF compared to wild type (WT) mice. While the magnitude of maximal alcohol-stimulated CBF increases did not differ between mice, the time to maximal CBF was significantly decreased in the DDAH mice versus WT mice treated with alcohol (*P < 0.05). No significant differences in CBF were observed in control, media-incubated tracheal rings. Bars represent SEM of triplicate separate experiments (*n* = 3).

Because ADMA is degraded by the action of dimethylaminohydrolase (DDAH) into L-citrulline, we hypothesized that overexpression of DDAH enhances alcohol stimulation of CBF. To test this hypothesis, we determined the impact of elevated DDAH levels on alcohol-stimulated NO action with regard to PKA activation and CBF in ciliated epithelium from mouse tracheal rings derived from transgenic mice overexpressing DDAH-I, ADMA-degrading enzyme. Compared to wild type mice, alcohol more rapidly stimulated CBF in the tracheal cilia of DDAH overexpressing mice. Alcohol-stimulated CBF in the DDAH mice at 15–35 min compared to 40–50 min in the wild type mice (Figure 4). Furthermore, the peak magnitude of enhanced CBF for DDAH mice at 30 min was significantly increased over the peak magnitude of wild type mice at 1 hr (data not shown). These results show that high levels of DDAH enhance the alcohol-stimulated cilia response by facilitating potential NOS activation in response to alcohol.

### 3.5. Alcohol-Stimulated Increases in Lung Slice Airway CBF and PKA Are Enhanced in DDAH Mice

**Figure 5:**
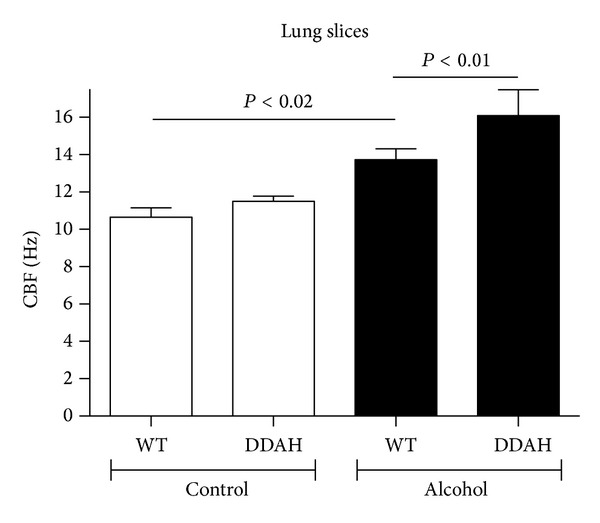
Alcohol-stimulated increases in lung slice airway CBF are enhanced in DDAH mice. Precision-cut lung slices from both wild type (WT) and dimethylaminohydrolase (DDAH) expressing mice were treated with alcohol, and their maximal CBF stimulation peaks were recorded. Alcohol (EtOH; 100 mM) significantly stimulated CBF in slices from both mice. Alcohol-stimulated CBF was significantly enhanced in DDAH (30 min) versus WT lung slices (1 hr). Bars represent SEM of triplicate separate experiments (*n* = 3).

**Figure 6:**
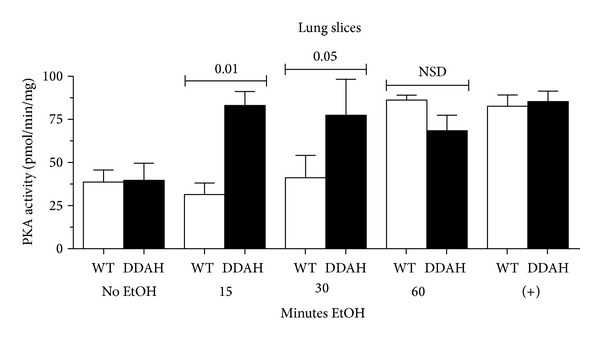
Alcohol-stimulated increases in lung slice PKA activity are enhanced in DDAH mice. Precision-cut lung slices from both wild type (WT) and dimethylaminohydrolase (DDAH) expressing mice were treated with alcohol, and their maximal PKA activity was recorded. Alcohol (EtOH; 100 mM) significantly stimulated PKA in slices from both mice at 1 hr. Alcohol-stimulated PKA was earlier in DDAH (15–30 min) versus WT lung slices (1 hr). Positive control (+) represents a 30 min treatment with 10 *μ*M 8Br-cAMP. Bars represent SEM of triplicate separate experiments (*n* = 3).

Previously, we reported a precision-cut lung slice model for measuring CBF changes [15]. Similar to tracheal ring CBF, alcohol rapidly (1 hr) stimulates increases in mouse lung slice airway CBF (Figure 5). We hypothesized that lung slices made from DDAH overexpressing mice would have enhanced CBF and PKA responses to alcohol. Indeed, CBF from DDAH overexpressing mice not only responds to 100 mM alcohol with stimulated increases in CBF, but also demonstrates a significant (*P* < 0.01) increase in CBF response versus wild type lung slices treated with alcohol. As with tracheal rings, the time of maximal CBF in lung slices is enhanced in DDAH mice as compared to wild type mice (30 min versus 1 hr). Similar to isolated BBEC, lung slice PKA was significantly (*P* < 0.05) increased by 1 hr treatment with 100 mM alcohol in wild type mice (Figure 6). In lung slices from DDAH mice, the time of PKA activation was significantly enhanced as compared to alcohol-stimulated wild type lung slices at 15 and 30 min. It was not until 1 hr that alcohol-stimulated PKA activity in wild type mice became equivalent to that of DDAH mice. These data demonstrate that airway epithelial-localized DDAH can function to enhance a NO-mediated cilia stimulation response (both in terms of increased PKA activity and CBF) in the preserved structure of the lung slice as well as the individual bronchial epithelial cell. Collectively, these data demonstrate that the action of ADMA regulates CBF in concert with the action of DDAH to affect the transitional impact of alcohol on cilia from initial CBF stimulation toward the eventual uncoupling of NO-mediated cilia stimulation observed with chronic alcohol desensitization of mucociliary clearance.

## 4. Discussion

Our findings demonstrate that ADMA, an endogenous inhibitor of NO, is highly expressed in the lung and is particularly localized to the ciliated airway epithelium. Pretreatment with ADMA blocks alcohol-stimulated increases in PKA activity and cilia beating in *in vitro *exposed bovine cell cultures. Mice that overexpress DDAH, an endogenous enzyme that degrades ADMA, demonstrate an enhanced PKA and CBF response in their tracheal ring ciliated epithelium to acute alcohol treatment. Similarly, mice that overexpress DDAH demonstrate an enhanced PKA and CBF response in their lung slices to acute alcohol treatment. These data suggest that ADMA is an endogenous regulator for the action of NO in response to alcohol exposure in the airways. ADMA may represent a potential pathway by which alcohol-elevated NO levels are reduced, PKA activity decreased, and stimulated cilia beating returned to baseline levels. 

**Figure 7:**
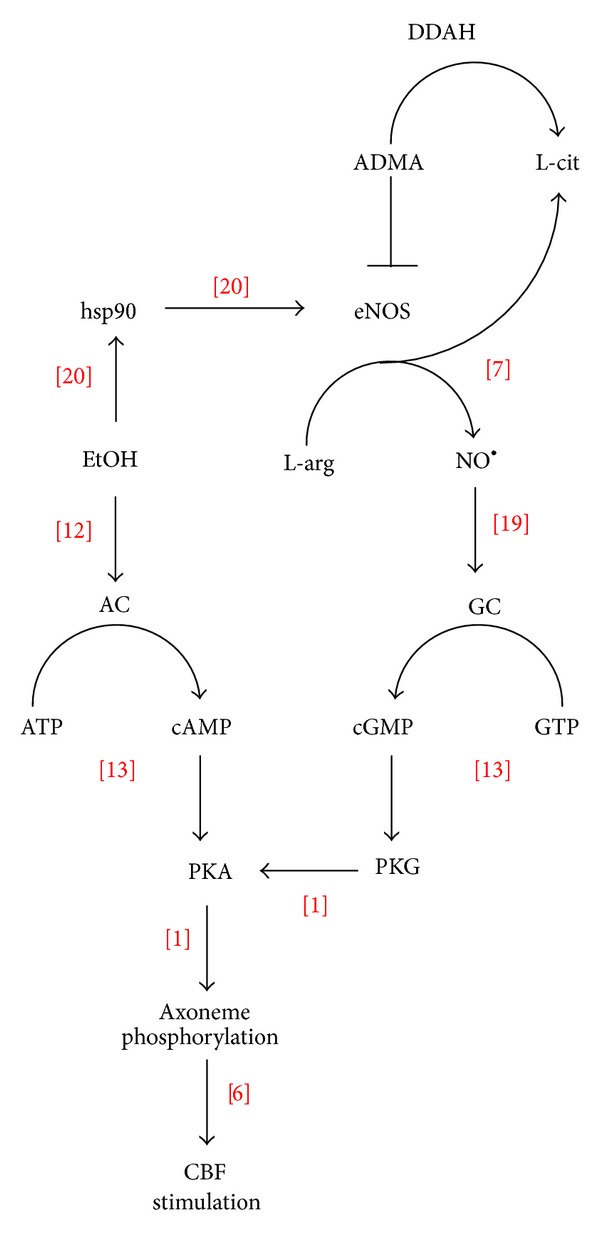
Proposed model of ADMA action on alcohol-stimulated cilia beating. Alcohol (EtOH) rapidly and transiently stimulates cilia beat frequency (CBF) by both activating the alcohol-sensitive adenylyl cyclase isoform 7 (AC) and increasing the phosphorylation of heat shock protein 90 (hsp90) leading to the increased binding of hsp90 and the activation of nitric oxide synthase (eNOS). Nitric oxide (NO^•^) activates soluble guanylyl cyclase (GC) leading to a cGMP-dependent protein kinase (PKG) activation required for alcohol-induced activation of cAMP-dependent protein kinase (PKA). Asymmetric dimethylarginine (ADMA) functions to negatively regulate alcohol stimulation of eNOS, except under the conditions of elevated dimethylarginine dimethylaminohydrolase (DDAH) whereby ADMA is converted to L-citrulline (L-cit).

Alcohol uniquely requires both NO [7] and cyclic nucleotide elevation [13] in order to stimulate CBF (Figure 7). An alcohol-sensitive soluble adenylyl cyclase [12] and guanylyl cyclase are all colocalized to what is termed the ciliary metabolon [19]. Without the generation of NO [1] and the activation of PKG [1], alcohol cannot activate PKA, a necessary kinase for ethanol-stimulated increases in CBF [6]. Recently, it was demonstrated that brief alcohol treatment leads to heat shock protein 90 (HSP90) phosphorylation and binding to eNOS in the apical region of the ciliated airway epithelium [20]. This binding and chaperone function of HSP90 may serve to activate eNOS in the ciliated cell. The action of ADMA may serve as an “off switch” to this eNOS activation, thus leading to the eventual return to baseline CBF levels observed shortly after alcohol treatment. Indeed, evidence exists that ADMA is capable of blocking HSP90 [21]. ADMA as a negative regulator of NO-activated CBF is supported by the inhibitory action of exogenous ADMA on alcohol-stimulated CBF and the enhancement of alcohol-stimulated CBF by the inhibitor of ADMA, DDAH. Both ADMA and DDAH are highly expressed in the airway epithelium.

The lung has been established as a major source for ADMA [9], and DDAH2 has been localized in the bronchial epithelium of mice [10, 18]. Arginine methylation of cellular proteins is catalyzed by protein arginine methyltransferases (PRMT). The expression of PRMT isoforms in the lung has been demonstrated to be primarily localized to the bronchial and alveolar epithelium [22]. It has been proposed that a delicate balance between ADMA-metabolizing enzymes is disturbed in bronchial epithelium during acute airway injury, potentially causing increased nitrosative stress in the form of ADMA-induced peroxynitrite production [10]. ADMA levels have been reported to be decreased in the plasma of individuals with alcohol use disorders in a recent paper by Frieling et al. [23]. However, in many disease states associated with alcohol abuse (such as hepatitis and cirrhosis), plasma ADMA levels have been demonstrated to be elevated in alcohol abuse [24, 25]. Thus, there is no clear consensus on the systemic ADMA levels after chronic alcohol consumption. There have been no specific lung tissue measurements of ADMA after alcohol consumption in humans reported. Acute consumption of alcohol results in elevated lung NO in the lung. Studies have not been reported in the alcoholic human lung, but chronic alcohol feeding results in the uncoupling of this NO response in rodents as ADMA levels are elevated, and stimulated NO levels are decreased [26, 27].

By itself, ADMA has been shown to have no effect on cilia beating in rat tracheal epithelium at concentrations up to 1 mM [28]. Superphysiologic concentrations (10 mM) resulted in a small, nonsignificant decrease in CBF. In addition, ADMA alone did not alter IL-8, RANTES, or TNF release from BEAS-2B. Our studies examining the effects of ADMA on mouse tracheal epithelial explant rings are in agreement with those of Galal et al. as we furthermore observed small decreases in CBF at the 10 mM ADMA dose [28]. These findings support the concept that ADMA does not impact cilia bioreactivity in the absence of NOS modulation by another agent such as alcohol. While nebivolol-induced degradation of ADMA levels through the elevation of DDAH-2 expression has been reported in endothelial cells [29], we found that nebivolol does not impact baseline CBF, nor does it enhance cilia responsiveness to alcohol (data not shown). This may be due to differences in cell type expression of DDAH-2 in response to nebivolol as transgenic mice overexpressing DDAH clearly enhance the alcohol stimulation of CBF.

ADMA has already been implicated in the pathogenesis of lung disease. Elevated ADMA exacerbates airway inflammation [18] and modifies lung function [30] in mouse models. Lung ADMA was elevated in a mouse model of allergic asthma, and the exogenous administration of inhaled ADMA to normal mice resulted in airway hyperresponsiveness to methacholine challenge, suggesting that ADMA is increased in asthma [31]. ADMA levels in the exhaled breath condensate of asthmatic children were significantly higher compared to those of healthy controls regardless of whether asthmatic children were on inhaled steroid treatment [32]. Alternately, plasma levels of ADMA were found to be significantly lower in allergic pediatric mild asthmatic patients compared to healthy subjects [33]. Statins may function to modulate asthmatic lung injury via the modulation of ADMA. Using an alveolar epithelial cell model, ADMA and inducible nitric oxide synthase were reduced by simvastatin, but eNOS was increased [34]. Increased severity of lung injury after *P*. *aeruginosa* sepsis was associated with elevated ADMA concentrations [35]. Impaired mucociliary clearance due to elevated ADMA may be an additional mechanism contributing to the severity of acute lung injury in *P*. *aeruginosa* sepsis.

Although elevated ADMA does not appear to impair or slow CBF as evidenced by our data, ADMA may function to slow cilia in concert with a cilia toxin in an opposite manner to how DDAH enhances CBF only under conditions of a NOS activating agent such as alcohol. For example, Wu et al. [36] showed that ADMA increased protein kinase C (PKC) while DDAH decreased PKC expression in response to ischemia/reperfusion injury with the opposite effects on NO levels in the lung. Recently, we have identified that cilia *slowing* is actively regulated through PKC epsilon in response to many agents that are known to have a cilia slowing effect [15]. Perhaps, in the presence of elevated ADMA, an enhanced cilia slowing response would be produced under injury conditions by a PKC epsilon-activating cilia toxin. Thus, the enhancement of proper innate defense against inhaled pathogens might focus on therapeutic agents that either decrease ADMA or increase DDAH in the airway epithelium.
